# Prevalence and risk factors for selected canine vector-borne diseases in Greece

**DOI:** 10.1186/s13071-019-3543-3

**Published:** 2019-06-03

**Authors:** Athanasios Angelou, Athanasios I. Gelasakis, Natalia Verde, Nikola Pantchev, Roland Schaper, Ramaswamy Chandrashekar, Elias Papadopoulos

**Affiliations:** 1 Laboratory of Parasitology and Parasitic Diseases, School of Veterinary Medicine, Faculty of Health Sciences, Aristotle University of Thessaloniki, PO Box: 393, 54124 Thessaloniki, Greece; 2 Laboratory of Farm Animal Anatomy and Physiology, Department of Animal Science and Aquaculture, School of Agricultural Production, Infrastructure and Environment, Agricultural University of Athens, 11855 Iera Odos, Greece; 3 Laboratory of Photogrammetry and Remote Sensing, Department of Cadastre, Photogrammetry and Cartography, Faculty of Rural and Surveying Engineering, Aristotle University of Thessaloniki, 54124 Thessaloniki, Greece; 4 IDEXX Laboratories, 71636 Ludwigsburg, Germany; 5 Bayer Animal Health GmbH, 51368 Leverkusen, Germany; 6 IDEXX Laboratories Inc., Westbrook, ME 04092 USA

**Keywords:** Vector-borne, Dogs, Risk factors, Climate, Altitude, Temperature, Rainfall, Greece

## Abstract

**Background:**

Canine vector-borne diseases (CVBDs) represent a wide group of diseases of major significance for canine health. In addition to their veterinary importance, many of these diseases are of great zoonotic concern, posing a risk of potential transmission to humans. To date, there has been scant knowledge regarding the prevalence, distribution and risk factors of CVBDs in Greece. Therefore, the objectives of the present study were to update the current knowledge on the seroprevalence of *Dirofilaria immitis*, *Anaplasma* spp., *Ehrlichia* spp. and *Borrelia burgdorferi* (*sensu lato*) in dogs in Greece and, furthermore, to assess possible environmental and any other risk factors associated with these infections. A total of 1000 apparently healthy and randomly selected dogs, presented in veterinary clinics, were involved at the national level (*n* = 66 municipalities). Serum samples were obtained from each individual dog and were tested using the SNAP^®^ 4Dx^®^ Plus kit from IDEXX Laboratories. Possible risk factors were assessed using binary regression models, including dogs’ lifestyle, climatological parameters and the altitude of the region.

**Results:**

Overall, 21.8% (95% CI: 19.4–24.5%) of the sampled dogs were found to be seropositive to at least one of the four pathogens examined. The most prevalent pathogen was *Ehrlichia* spp. (12.5%, 95% CI: 10.6–14.7) followed by *D. immitis* (9.0%, 95% CI: 7.8–11.5) and *Anaplasma* spp. (6.2%, 95% CI: 4.9–7.9). The lowest prevalence (0.1%) was recorded for *B. burgdorferi* (*s.l.*) where only one dog was found to be positive. Among the examined risk factors, low mean temperature was found to increase the prevalence of *Ehrlichia* spp. (*P* ≤ 0.001) and *Anaplasma* spp. (*P* ≤ 0.001), while low minimum temperature increased the prevalence of *D. immitis* (*P* ≤ 0.001). In addition, low total annual rainfall had an effect of the prevalence of *Ehrlichia* spp. (*P* ≤ 0.01). Altitude also had a significant effect on the prevalence of *D. immitis* (*P* ≤ 0.05) and *Anaplasma* spp. (*P* ≤ 0.01).

**Conclusions:**

To our knowledge, this is the first large-scale seroepidemiological study of CVBDs in Greece. It has been evidenced that environmental factors such as temperature, rainfall and altitude can influence the prevalence and distribution of CVBDs.

## Background

Canine vector-borne diseases (CVBDs) can significantly impact canine health status. The etiology of CVBDs is wide, including a variety of pathogens, i.e. protozoans, helminths, bacteria and viruses, all transmitted by hematophagous arthropods such as mosquitoes, ticks, fleas, lice and phlebotomine sandflies [1, 2]. CVBDs have a wide range of clinical manifestation, from asymptomatic cases to severe health implications, depending on the pathogenicity of the specific causative agent and the presence of single or co-infections, which complicates their diagnosis, control and treatment for veterinary practitioners [3, 4]. Moreover, animals with subclinical CVBDs infections are more susceptible to other infectious diseases [5, 6]. In addition to their veterinary importance, many of these diseases are of major zoonotic concern, posing a risk of potential transmission to humans [3, 7]. Therefore, the control of these diseases is a challenging field with evident benefits for both animal and public health [3, 8]. In this respect, *Ehrlichia canis*, *Dirofilaria immitis*, *Anaplasma phagocytophilum* and *Borrelia burgdorferi* are of great concern [4].

During recent decades, the worldwide distribution of CVBDs has been constantly changing [9]. This is attributed to a plethora of anthropogenic factors, including climate change, globalization, international transportation and trade, and the rapid growth of human, canine and wildlife reservoir populations [10]. Climate is a substantial factor considering the survival and spread of arthropod vectors and subsequently the distribution of CVBDs [9]. In particular, arthropod vectors and their life-cycles are strongly affected by environmental temperature. Apart from the life-cycle of vectors, the reproduction and survival rate of the parasitic and viral agents inside vectors and definitive hosts have also been found to be affected by environmental temperature [11].

Vectors (e.g. *Aedes albopictus*) and pathogens (e.g. *Leishmania infantum*, *D. immitis*) could expand or (re-)emerge due to climate change [12–15]. In Greece, according to the Intergovernmental Panel of Climate Change (IPCC), the climate is expected to evolve towards more tropical climates characterized by an extended dry and hot season [16], and this may affect the distribution of CVBDs [17] in the country.

According to relevant studies, many CVBDs have been reported in canine populations [18, 19], as well as in human populations in Greece [20–22]. However, the majority of the fore-mentioned studies have been focused on single species of pathogens and examined the situation in a restricted area and number of animals. Furthermore, none of these studies have assessed the relationship between climate and the seroprevalence of CVBDs in the country.

The aim of this study was to update the current state of knowledge on the seroprevalence of *Ehrlichia* spp., *D. immitis*, *Anaplasma* spp. and *B. burgdorferi* (*s.l.*), in Greece. In addition, we studied the effects of environmental and other risk factors on the seroprevalence of CVBDs in Greece.

## Methods

### Study area

At least one representative area from each municipality of the country was selected for sampling. This resulted in 66 locations similarly-spaced along the north-south and the east-west axes of the country for the sample collection. Among these areas there was a significant diversity of geographical and climate characteristics such as altitude, longitude, latitude, relative humidity, environmental temperature, total annual rainfall and wind speed.

### Animals and sample collection

**Fig. 1:**
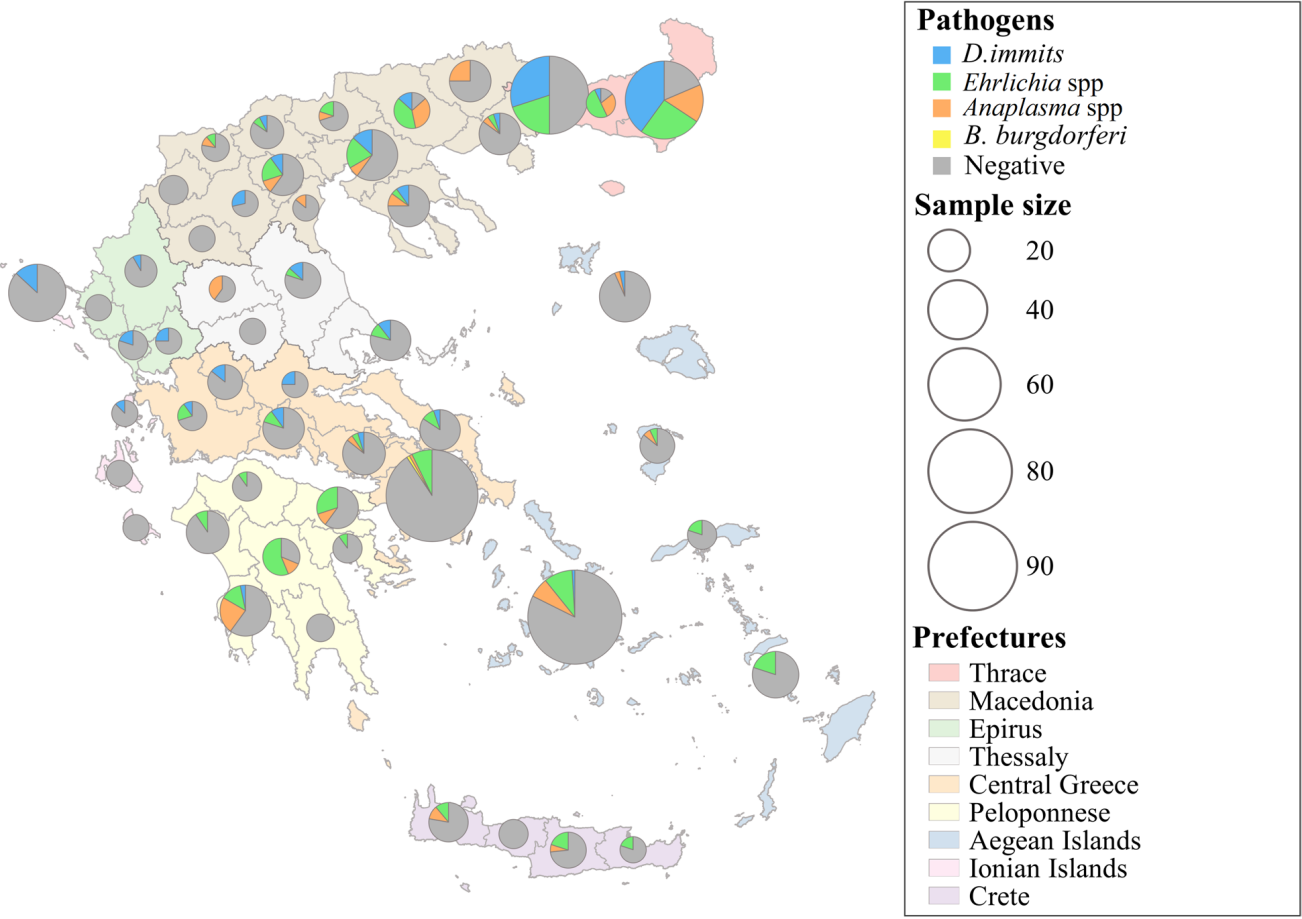
Geographical distribution of seropositive dogs for CVBDs in Greece

An overall population of 1000 dogs from 66 municipalities was included in the survey: 189 (18.9%) dogs from central Greece; 188 (18.8%) from Macedonia; 181 (18.1%) from Aegean Islands; 150 (15.0%) from Thrace; 116 (11.6%) from Peloponnese; 55 (5.5%) from Ionian Islands; 48 (4.8%) from Crete; 44 (4.4%) from Thessaly; and 29 (2.9%) dogs from Epirus (Fig. 1).

All sampled dogs were randomly selected and apparently healthy. A blood sample was collected from each individual dog when visiting the veterinary clinic for annual vaccination or routine inspection. Blood was collected from the cephalic or jugular vein into vacutainer tubes without anticoagulant and stored in the refrigerator for a maximum of 24 h until centrifugation. Samples were centrifuged at 1300–1800×*g* for 20 min, and then serum was separated from the clot. Serum samples were stored at − 20 °C until further assayed.

After sample collection, data regarding the characteristics of the dogs were collected using a structured, case-specific questionnaire including information about age, sex, breed, lifestyle (outdoors, indoors) of the dog and information about the last antiparasitic treatment (endo- and/or ectoparasiticides active against vectors or vector-borne pathogens, i.e. milbemycin, fipronil, permethrin, etc.), as presented in Table 1. All dogs participating in the study were apparently healthy at physical examination, not infested with ectoparasites and older than 6 months. The study lasted one year, from the 1st of January 2016 until the 31st of December 2016.

**Table 1:** Distribution of the dog population (*n* = 1000 dogs) according to the studied parameters

Parameter	Level	No. of studied dogs
Age (years)	< 1	69
	1–3	374
	3–5	279
	> 5	278
Sex	Male	468
	Female	532
Lifestyle	Outdoors	807
	Indoors	193
Breed	Crossbreeds	648
	English Setter	79
	German Shepherd	49
	Other purebred breeds	224
Last antiparasitic treatment (months passed)	No treatment	290
	< 3	457
	3–6	173
	6–12	54
	> 12	26
Mean environmental temperature (°C)	> 15.9	781
	≤ 15.9	219
Minimum environmental temperature (°C)	> − 5.5	665
	≤ − 5.5	335
Maximum environmental temperature (°C)	> 38.3	348
	≤ 38.3	652
Mean humidity (%)	> 69.7	456
	≤ 69.7	544
Total annual rainfall (mm)	> 554	701
	≤ 554	299
Mean wind speed (knots)	> 10.7	127
	≤ 10.7	873
Altitude (masl)	0–100	811
	101–400	123
	401–900	66

*Abbreviation*: masl, metres above sea level

### Climatological and altitude data

For each of the studied regions climatological data were collected including mean, minimum and maximum environmental temperature (°C), mean humidity (%), total annual rainfall (mm) and mean wind speed (knots). Meteorological data were acquired from two climatological databases, namely the Hellenic National Meteorological Services (HNMS) and the “Meteo View” platform. The HNMS is the official government agency responsible for the weather forecast and observations at the national level. The “Meteo View” platform is a web-based geographical information system (GIS) tool created and hosted by the “Meteo” weather service. It was made for monitoring and analyzing the meteorological station network set up by Meteo, the Institute of Environmental Research and Sustainable Development (IERSD) and the National Observatory of Athens (NOA). For each studied region, the nearest meteorological station was selected, and its data were used for further analyses. Mean values for each metric were obtained from HNMS and supplemented by the “Meteo View” when it was necessary. The mean values obtained from these metrics were as follows: 15.9 °C, −5.5 °C and 38.3 °C for mean, minimum and maximum environmental temperature, respectively, 69.7% for mean relative humidity, 554 mm for total annual rainfall and 10.7 knots for mean wind speed.

The altitude data of the studied regions were derived from a digital elevation model (DEM) and data collected by NASA’s Shuttle Radar Topography Mission (SRTM) which provides an accuracy of approximately ± 1.73 m. This accuracy level has been considered sufficient for similar studies and has already been used in order to extract geographical information [23, 24]. The altitude measurements were taken from points on the map which represented the exact place of sampling. Three altitude-zones were considered: 0–100 metres above sea level (masl); 101–400 masl; and 401–900 masl.

### Serological analysis

Serum samples were tested using a rapid enzyme-linked immunosorbent assay (ELISA) kit (SNAP^®^ 4Dx^®^ Plus Test Kit, IDEXX Laboratories, Inc, Westbrook, ME, USA), according to the manufacturer instructions. Two spots are impregnated respectively with a specific peptide antigen of *B. burgdorferi* (*s.l.*) (C6 peptide, derived from the IR6 region within the *Borrelia* membrane protein VlsE; [25]) and *E. canis* (peptides from p30 and p30-1 outer membrane proteins; cross-reactive with *Ehrlichia chaffeensis*; [26]). The device additionally detects antibodies to *E. ewingii* (peptide derived from p28 outer surface protein family; [27]). The *D. immitis* analyte is derived from two antibodies (one for capture and the other for detection) specific to heartworm antigens, which are primarily produced by adult females. This in-clinic test detects specific antibodies to *A. phagocytophilum*/*A. platys* (peptide from the major surface protein p44/MSP2; [27]). In contrast to the whole-cell antigen present on *A. phagocytophilum* IFA slides, no genus level cross-reaction between *Anaplasma* and *Ehrlichia* is observed within this device based on the utilization of specific peptides [28]. The respective sensitivity and specificity of the test system are 93.2 and 99.2% for *A. phagocytophilum*, 89.2 and 99.2% for *A. platys*, 96.7 and 98.8% for *B. burgdorferi* (*s.l.*), 97.8 and 92.3% for *E. canis*, and 98.9 and 99.3% for *D. immitis*. Furthermore, a cross-reactivity of *E. canis* antigens with anti-*E. chaffeensis* antibodies was shown. Due to documented cross-reactivity between *A. phagocytophilum* and *A. platys*, as well as the reactivity to *E. canis*, *E. chaffeensis* and *E. ewingii* [27], we refer to *Anaplasma* spp. and *Ehrlichia* spp. in the results of the present study (as isolates were not identified to the species level).

### Data handling and statistical analysis

Data were recorded in a Microsoft Excel spreadsheet and imported into IBM SPSS Statistics v.23.0. for the subsequent statistical analyses. The prevalence of parasitic infections was defined as the proportion of positive animals to the total number of the examined animals and the 95% confidence intervals (CI) of the prevalence values were estimated using the Wilson score interval method. Stepwise binary logistic regression models were used to assess the effects of possible risk factors [age, sex, habitat, use, last antiparasitic treatment, altitude, environmental temperature (mean, minimum and maximum), mean humidity, total annual rainfall and wind speed] on the likelihood that a dog is infected with *Ehrlichia* spp., *D. immitis* and *Anaplasma* spp.

A forward stepwise selection procedure of the variables was followed and only predictors which had a significant effect (*P* ≤ 0.05) on each individual parasitic infection were used for the final models. According to this, the likelihood of (i) *Ehrlichia* spp., (ii) *D. immitis*, and (iii) *Anaplasma* spp. infection were estimated using as predictors the regression coefficients of (i) habitat use, last antiparasitic treatment, mean temperature and total annual rainfall, (ii) altitude, last antiparasitic treatment and minimum temperature, and (iii) altitude, last antiparasitic treatment and mean temperature.

Statistical significance of individual predictors was tested using the Wald Chi-square statistic of their regression coefficients (βs). The Hosmer–Lemeshow (H–L) test, Cox and Snell *R*^2^ and Nagelkerke *R*^2^ indices were also calculated to assess the goodness-of-fit for each individual model.

## Results

The distribution of the sampled dogs according to climatological data and the altitude of the studied areas are summarized in Table 1.

### Seroprevalence of CVBDs in the studied dog population

Overall, 21.8% (218/1000, 95% CI: 19.4–24.5%) of the sampled dogs were found to be seropositive to at least one of the four pathogens examined in this present study (Table 2). Moreover, the proportion of dogs that were seropositive against one, two or three parasites was 16.5% (165/1000, 95% CI: 14.3–18.9%), 4.7% (47/1000, 95% CI: 3.6–6.2%) and 0.6% (6/1000, 95% CI: 0.3–1.3), respectively (Table 2). Overall, the most prevalent canine vector-borne pathogen was *Ehrlichia* spp. (12.5%, 125/1000, 95% CI: 10.6–14.7%) followed by *D. immitis* (9.0%, 90/1000, 95% CI: 7.8–11.5%) and *Anaplasma* spp. (6.2%, 62/1000, 95% CI: 4.9–7.9%); the lowest prevalence was recorded for *B. burgdorferi* (*s.l.*) where only one dog was found to be positive (0.1%). The detailed epizootiological map deriving from our results is presented in Fig. 1.

**Table 2:** Number and prevalence of canine vector-borne infections and co-infections in the studied dog population (*n* = 1000 dogs) in Greece

Pathogen	No. of infected dogs	Prevalence (%)
*Dirofilaria immitis*	59	5.9
*Ehrlichia* spp.	79	7.9
*Anaplasma* spp.	27	2.7
*Dirofilaria immitis + Ehrlichia* spp.* + Anaplasma* spp.	5	0.5
*Dirofilaria immitis + Ehrlichia* spp.	19	1.9
*Dirofilaria immitis + Anaplasma* spp.	7	0.7
*Ehrlichia* spp.* + Anaplasma* spp.	21	2.1
*Ehrlichia* spp.* + Anaplasma* spp.* + Borrelia burgdorferi* (*s.l*.)	1	0.1
Total	218	21.8

### Risk factors associated to CVBD seroprevalence

Table 3 summarizes the risk factors for CVBDs and their effects on seropositivity status. For *Ehrlichia* spp., animals that lived outdoors were more likely to be seropositive to *Ehrlichia* spp. in comparison with those living indoors (*P* = 0.013, OR: 2.3, 95% CI: 1.2–4.4). Moreover, companion dogs were more likely to be seropositive for *Ehrlichia* spp. than hunting dogs (*P* = 0.002, OR: 2.6, 95% CI: 1.4–4.6). Additionally, the likelihood of *Ehrlichia* spp. seropositivity was higher for the animals that have not received antiparasitic treatment (endo- and/or ectoparasiticides active against vectors or vector-borne pathogens, i.e. milbemycin, fipronil, permethrin, etc.) for 12 months prior to sampling, when compared to those that had never received antiparasitic treatment (*P* = 0.03, OR: 2.7, 95% CI: 1.1–6.7), those that had received treatment in the last 6 to 12 months (*P* = 0.007, OR: 5.6, 95% CI: 1.6–19.6), 3 to 6 months (*P* = 0.001, OR: 5.1, 95% CI: 1.9–13.9) and less than 3 months (*P* = 0.0001, OR: 5.6, 95% CI: 2.3–13.9). Regarding the climatological conditions, the likelihood of *Ehrlichia* spp. seropositivity was higher for dogs living in areas with a mean temperature < 15.9 °C (*P* = 0.001, OR: 2.2, 95% CI: 1.4–3.5) in comparison to dogs living in areas with mean temperature ≥ 15.9 °C. Similarly, dogs that were living in areas with lower total annual rainfall were more likely to be seropositive to *Ehrlichia* spp. when compared to those animals that were living in areas with higher total annual rainfall (*P* = 0.004, OR: 2.1, 95% CI: 1.3–3.5). In the case of *D. immitis* infection, dogs that were living in areas with an altitude of 0–100 masl were more likely to be infected when compared to dogs living in areas with an altitude of 401–900 masl (*P* = 0.019, OR: 3.2, 95% CI: 1.2–8.5). Additionally, the likelihood of *D. immitis* seropositivity was higher for the animals that had not received antiparasitic treatment for more than 12 months comparing to those that had received treatment in the last 3 months (*P* < 0.0001, OR: 5.8, 95% CI: 2.2–15.1) and those that had received treatment in the last 3 to 6 months (*P *< 0.0001, OR: 7.6, 95% CI: 2.5–23.8). Moreover, the likelihood of *D. immitis* seropositivity was higher for dogs living in areas with a minimum temperature < −5.5 °C in comparison to dogs living in regions with a minimum temperature ≥ −5.5 °C (*P* < 0.0001, OR: 4.5, 95% CI: 2.7–7.5). The effects of the risk factors on *Anaplasma* spp. seropositivity status are presented in Table 3. The likelihood of *Anaplasma* spp. seropositivity was higher for dogs living in areas with a mean temperature < 15.9 °C when compared to those dogs living in areas with mean temperature ≥ 15.9 °C (*P* < 0.0001, OR: 4.2, 95% CI: 2.3–7.8). Moreover, dogs that were sampled from areas with an altitude of 0–100 masl were more likely to be seropositive to *Anaplasma* spp. in comparison with dogs living in areas with an altitude of 401–900 masl (*P* = 0.010, OR: 7.1, 95% CI: 1.6–31.5). Finally, the likelihood of *Anaplasma* spp. seropositivity was higher for the animals that had never received any kind of antiparasitic treatment (endo- and/or ectoparasiticides active against vectors or vector-borne pathogens, i.e. milbemycin, fipronil, permethrin, etc.) compared to the those that had received antiparasitic treatment in the last 3 to 6 months (*P* = 0.013, OR: 3.5, 95% CI: 1.3–9.3).

**Table 3:** *P*-values, odds ratios and 95% CI for odds ratios, of the predictor variables used in *Ehrlichia* spp., *Dirofilaria immitis* and *Anaplasma* spp. models

Variable	*P*-value	Odds ratio	95% CI	
*Ehrlichia* spp.				
Habitat, outdoors	0.013	2.287	1.193–4.383	
Habitat, indoors	Ref.			
Use, hunting	0.002	0.392	0.217–0.708	
Use, companion	Ref.			
Last antiparasitic treatment, none	0.030	0.370	0.150–0.910	
Last antiparasitic treatment < 3 months	< 0.0001	0.179	0.072–0.442	
Last antiparasitic treatment 3–6 months	0.001	0.195	0.072–0.525	
Last antiparasitic treatment 6–12 months	0.007	0.178	0.051–0.625	
Last antiparasitic treatment > 12 months	Ref.			
Mean temperature < 15.9 °C	0.001	2.171	1.364–3.457	
Mean temperature ≥ 15.9 °C	Ref.			
Total annual rainfall < 554 mm	0.004	2.114	1.267–3.528	
Total annual rainfall ≥ 554 mm	Ref.			
Constant	0.001	0.151		
*Dirofilaria immitis*				
Altitude 0–100 masl	0.019	3.213	1.210–8.534	
Altitude 101–400 masl	0.556	1.463	0.412–5.202	
Altitude 401–900 masl	Ref.			
Last antiparasitic treatment, none	0.072	0.442	0.181–1.077	
Last antiparasitic treatment < 3 months	< 0.0001	0.173	0.066–0.457	
Last antiparasitic treatment 3–6 months	< 0.0001	0.131	0.042–0.407	
Last antiparasitic treatment 6–12 months	0.746	0.837	0.286–2.449	
Last antiparasitic treatment > 12 months	Ref.			
Minimum temperature < −5.5 °C	< 0.0001	4.469	2.667–7.489	
Minimum temperature ≥ −5.5 °C	Ref.			
Constant	0.001	0.151		
*Anaplasma* spp.				
Altitude 0–100 masl	0.010	7.126	1.614–31.469	
Altitude 101–400 masl.	0.311	2.395	0.443–12.958	
Altitude 401–900 masl	Ref.			
Last antiparasitic treatment < 3 months	0.104	0.598	0.322–1.111	
Last antiparasitic treatment 3–6 months	0.013	0.288	0.108–0.772	
Last antiparasitic treatment 6–12 months	0.085	0.168	0.022–1.281	
Last antiparasitic treatment > 12 months	0.998	0.000	0.000	
Last antiparasitic treatment, none	Ref.			
Mean temperature < 15.9 °C	<0.0001	4.242	2.302–7.820	
Mean temperature ≥ 15.9 °C	Ref.			
Constant	<0.0001	0.012		

*Abbreviations*: masl, metres above sea level; CI, confidence interval; Ref., reference category

## Discussion

CVBDs are of major importance for veterinarians as well as for public health practitioners and constitute a field of increased scientific interest worldwide. The primary aim of this multicentric study was to assess the seroprevalence of CVBDs in Greece and investigate possible relationships between these infections, climate conditions and altitude. To the best of the authors’ knowledge, this is the first study conducted in Greece in order to assess the seroprevalence of CVBDs in canine population, which includes a large sample derived from each prefecture of the country and thus generates valuable and resilient data.

Overall, according to our results the recorded prevalence of CVBDs in dogs from Greece was high, with 21.8% of the sampled dogs being seropositive to at least one of the tested pathogens. Specifically, *Ehrlichia* spp. (12.5%) were the most prevalent canine vector-borne pathogens followed by *D. immitis* (9.0%) and *Anaplasma* spp. (6.2%); the lowest prevalence was detected for *B. burgdorferi* (*s.l*.) (0.1%). Correspondingly, previous studies have reported high prevalence estimates of CVBDs in other European countries, even though various diagnostic methods were used. In neighboring countries, such as Bulgaria and northeastern Turkey, studies have demonstrated a high prevalence of CVBDs with overall percentages of 64.7 and 48.9%, respectively [25, 29], whereas in Italy and Romania the overall prevalence rates were comparatively lower, i.e. 10.3 and 11.3% respectively [30, 31]. Other studies from the Balkan Peninsula have indicated an overall prevalence of CVBDs ranging from 25.7% in Croatia to 25.1% in Albania [32, 33]. Higher prevalence rates have been observed in Spain (37.1%) [34] and Portugal (66%) [35]. In some of the aforementioned studies, the results reflect the simultaneous presence of other pathogens, i.e. *Leishmania* spp.

Among the examined vector-borne pathogens, *Ehrlichia* spp. was found to be the most common (12.5%). Ehrlichiosis, apart from its veterinary importance, is of public health concern since some *Ehrlichia* species can also infect humans [36, 37]. To the best of our knowledge, prior to the present study there were no data available regarding the prevalence of *Ehrlichia* spp. in canine populations in Greece. Contrarily, there are some clinical studies reporting several cases of *Ehrlichia* spp. natural infections in dogs [38–41]. According to our findings, the likelihood of seropositivity to *Ehrlichia* spp. was associated mainly with the lifestyle of dogs and the antiparasitic scheme followed by the owner. First, dogs that were living outdoors had a higher possibility of being seropositive to *Ehrlichia* spp. when compared to those living indoors (*c.*2.3 times, *P* ≤ 0.05). This result can be attributed to the fact that dogs living outdoors have an increased chance of being exposed to the natural environment of the brown dog tick *Rhipicephalus sanguineus* (*s.l.*), which is the main vector of this pathogen and also the dominant tick species in dogs in Greece [42]. Lifestyle seems to have a significant effect on the seroprevalence of *Ehrlichia* spp. but not on the seroprevalence of the other studied tick-borne pathogens. A possible explanation relies to the fact that *Ehrlichia* spp. was the most prevalent pathogen and therefore the likelihood of its transmission is probably higher than that of the other examined tick-borne pathogens. Secondly, we found that that companion dogs had a greater chance of being seropositive to *Ehrlichia* spp. when compared to hunting dogs (*c.*2.6 times, *P* = 0.002). Parameters that could explain this finding include age and health status of the studied dogs. Hunting dogs are working dogs and hence they are mostly young dogs with a good health status which is necessary for being capable of hunting and also better prophylactically protected. When they grow older usually, they are donated and afterwards they are kept as companion dogs. Companion dogs, on the other hand, do not have any limitations regarding age or health status. Furthermore, it was found that dogs that did not receive frequent antiparasitic treatment tended to be seropositive to *Ehrlichia* spp. when compared to the animals that received them on a regular basis. As expected, the present result indicates that animals that received few antiparasitic treatments (including ectoparasiticides, i.e. fipronil and permethrin) were more likely to be infested by ticks and therefore it underlines the necessity of prevention against ectoparasites. In addition to the individual parameters of the dog, we also assessed environmental parameters as confounding factors. Nonetheless, a confounding effect regarding the region of the collected samples could be possible. In any case, comparisons between the 66 municipalities included in the sampling campaign was not within the scope of the present study and hence we selected not to use the fixed effect of region for the statistical analyses. Instead, we decided to assess climatic conditions (which are significant descriptors of the region) as possible risk factors. Under this perspective, it is possible to enhance the applicability of the results in other cases and regions where a similar climate exists. Regarding climatological conditions, surprisingly, it was demonstrated that dogs that were living in areas with a mean temperature < 15.9 °C were more likely to be seropositive to *Ehrlichia* spp. than dogs living in areas with a mean temperature ≥ 15.9 °C (*c.*2.2 times, *P* = 0.001). The exposure to extreme cold temperatures has a significant harmful effect on the development of *R. sanguineus* (*s.l.*) [43]. However, in Greece the climate remains typical mild Mediterranean, even in regions with a mean temperature < 15.9 °C, which favors the development of ticks. Moreover, we found that there is an effect of the total annual rainfall in the seroprevalence of *Ehrlichia* spp.: dogs that were living in dry areas with a low total annual rainfall < 554 mm (*c.*2.1 times, *P* = 0.004) were more likely to be seropositive to *Ehrlichia* spp. when compared to the animals that were living in wetter areas with a total annual rainfall ≥ 554 mm. Similar results have been previously reported in a study in Brazil where dogs were more likely to be infested by *R. sanguineus* ticks in drier environments [44]. This could be linked to the fact that *R. sanguineus* ticks are better adapted to drier climates when compared to other tick species, such as *Ixodes ricinus*, which prefer wetter climates [45].

The second most frequent canine vector-borne pathogen detected in our study was *D. immitis* with an overall antigen seropositivity of 9.0%. *Dirofilaria immitis*, widely known as the canine heartworm, is a filarial nematode transmitted by mosquitoes (*Culex* spp., *Aedes* spp. and *Anopheles* spp.) acknowledged to be the causative agent of cardiopulmonary dirofilariosis in dogs (heartworm disease) [46]. *Dirofilaria immitis* can also cause pulmonary dirofilariosis in humans, thus resulting in public health implications [47]. Many European countries, including Greece, are enzootic for this disease [18, 48]. Previous surveys from Greece have reported similar results; however, these were not large-scale studies and cannot be considered representative of the situation at a national level [18, 49, 50]. Additionally, dirofilariosis has also been previously reported in humans in Greece, a fact that underscores its zoonotic potential in the country [51, 52]. Nowadays, *D. repens* attracts more scientific interest as an emerging zoonotic agent than *D. immitis.* It is likely that in the past *Dirofilaria* species in humans were not always correctly identified and remained underdiagnosed at species level [53]. According to our results, the likelihood of antigen detection of *D. immitis* was associated mainly with the last antiparasitic treatments (i.e. milbemycin, permethrinor, other antiparasitics with repellent or larvicide activity against *Dirofilaria* spp.) of the examined animals. It can be concluded that unprotected animals are more susceptible to vector-borne infections. Therefore, the demand for frequent and effective chemoprophylaxis, for all dogs, all year round and regardless of the occurrence of infection should be emphasized. Moreover, it should be stressed that prevention for *Dirofilaria* infections is essential from both a veterinary and public health perspective. Altitude was found to be associated with *D. immitis* infection: dogs living in regions with a low altitude of 0–100 masl were more susceptible to dirofilariosis compared to dogs living in areas with a high altitude (401–900 masl). Mountainous regions with high altitude have low mean temperatures all year round and fewer water catchments, wetlands and valleys, which makes them unfavorable for the development of mosquitoes and, hence, the spreading of the disease. Similar results indicating this inversely proportional relationship between altitude and distribution of a mosquito-borne disease have been reported for malaria [54]. The likelihood of *D. immitis* seropositivity was also associated with the minimum temperature of the region of origin of the studied dogs. Surprisingly, our results indicate that dogs that were living in areas with a minimum temperature < −5.5 °C had a higher probability of being *D. immitis-*antigen-positive in comparison to those that were living in areas with a minimum temperature ≥ −5.5 °C. However, it has already been stated from previous studies that there is a northward geographical distribution of *D. immitis* in Greece [18], something that corresponds to our results, as the majority of regions with low minimum temperature are located in the northern part of the country. This finding can be easily explained by the increased population of mosquitoes in the northern parts of Greece [55], where water retention sites, ponds and agricultural valleys are more common. In the present study, the association between climatological conditions, altitude and seroprevalence of canine dirofilariosis has been assessed for the first time in Greece. Notably, in the present study, cases of canine dirofilariosis were reported in the Cyclades Islands and Southern Peloponnese, thus indicating a possible spread southward.

The third examined canine vector-borne pathogen group were *Anaplasma* species*. Anaplasma plat*ys, transmitted by *R. sanguineus* (*s.l.*), infects the platelets and is considered as the etiological agent of infectious canine cyclic thrombocytopenia [56, 57]. Conversely, *A. phagocytophilum*, transmitted by *Ixodes ricinus* ticks, infects white blood cells, mainly neutrophils, causing granulocytic anaplasmosis in dogs [58]. Additionally, *A. phagocytophilum* could be pathogenic for humans with potential zoonotic implications [58]. Due to their molecular similarity and cross-reactions, it is almost impossible to differentiate *A. platys* and *A. phagocytophilum* using serological analysis [59] while mixed infections are possible [60]. It was found that the overall prevalence of *A. plat*ys and/or *A. phagocytophilum* seropositivity was 6.2%. Data regarding the seroprevalence of *Anaplasma* spp. in the canine population in Greece were scarce except for some sporadic reported cases of *A. phagocytophilum* infections [19, 61]. A recent study reported *A. plat*ys in *R. sanguineus* ticks in Greece [42]. There are some studies considering human anaplasmosis due to *A. phagocytophilum* highlighting the zoonotic potential of this disease in Greece [20, 62, 63]. According to the results obtained for *Ehrlichia* spp. and *D. immitis*, a negative correlation between the seropositivity to *Anaplasma* spp. and the time of the last antiparasitic (i.e. fipronil, permethrin, etc.) treatment of the dog was reported. This result underlines that unprotected animals are at a significant risk of acquiring any of the CVBDs. Moreover, the mean environmental temperature poses a significant risk factor for the seropositivity to *Anaplasma* spp. As is the case for *Ehrlichia* spp., mean environmental temperature acts as a crucial factor for the development and distribution of ticks thereby affecting the distribution of tick-borne diseases, including anaplasmosis. Altitude of a region significantly affects the likelihood of seropositivity to *Anaplasma* spp., which is consistent with a previous study, where it was indicated that canine anaplasmosis due to *A. phagocytophilum* is an altitude-dependent disease [64].

In the present study the seropositivity to the spirochete *B. burgdorferi* was assessed. *Borrelia burgdorferi* (*s.l.*) complex includes at least 19 species and is transmitted mainly by ticks of the genus *Ixodes* [26]. *Borrelia burgdorferi* is the primary agent that causes Lyme borreliosis in dogs and humans [65]. Dogs are naturally exposed to tick infestations, may act as reservoirs, and consequently play a significant role to the transmission of Lyme disease to humans [66]. Although Lyme borreliosis in humans has been previously reported in Greece [67, 68] this is the first time a seropositive dog for *B. burgdorferi* has been identified. It should be noted that the prevalence of infection detected in dogs is low. Correspondingly, the incidence of human borreliosis in Greece is the lowest among other European countries [69]. The above can be attributed to the fact that the main tick species that infests dogs in Greece is the brown dog tick (*R. sanguineus*), which does not transmit *B. burgdorferi*. On the other hand, *I. ricinus* that is the main vector of *B. burgdorferi* is very rare in Greece [42]. As expected, in northern countries of Europe where the prevalence of *I. ricinus* is high, the occurrence of Lyme borreliosis in dogs is higher.

## Conclusions

Our results suggest that animals living outdoors and receiving only occasionally or no antiparasitic treatments are prone to infections with canine vector-borne pathogens. These findings highlight the significance and the urgent need for appropriate antiparasitic treatments, especially for those dogs living mostly outdoors. Additionally, it is essential to underline that early diagnosis by using quick with high sensitivity and specificity diagnostic tests such as SNAP^®^ 4Dx^®^ Plus, as well as early intervention of CVBDs could improve the expected prognosis, especially in life-threatening diseases. Finally, our work represents the first attempt to demonstrate the relationships between the seroprevalence of CVBDs and climatological conditions, including altitude. Among the examined climatologic conditions, mean and minimum temperature, rainfall and altitude can influence the prevalence and distribution of CVBDs.

## Data Availability

Data supporting the conclusions of this article are included within the article.

## Abbreviations

CVBDs: canine vector-borne diseases
CI: confidence interval
*s.l.*: *sensu lato*
HNMS: Hellenic National Meteorological Services
masl: meters above sea level
